# Predictive factors of acute skin reactions to carbon ion radiotherapy for the treatment of malignant bone and soft tissue tumors

**DOI:** 10.1186/s13014-017-0927-4

**Published:** 2017-11-22

**Authors:** Yosuke Takakusagi, Jun-ichi Saitoh, Hiroki Kiyohara, Takahiro Oike, Shin-ei Noda, Tatsuya Ohno, Takashi Nakano

**Affiliations:** 1 Gunma University Heavy Ion Medical Center, 3-39-22, Showa-machi, Maebashi, Gunma 371-8511 Japan; 2 Department of Radiation Oncology, Gunma University Graduate School of Medicine, 3-39-22, Showa-machi, Maebashi, Gunma 371-8511 Japan

**Keywords:** Acute skin reactions, Carbon ions, Dermatitis, Malignant bone and soft tissue tumors, Predictive factors

## Abstract

**Background:**

The skin is considered a critical organ at risk in carbon ion radiotherapy (CIRT) for locally advanced malignant bone and soft tissue tumors (MBSTs). The predictive factors for acute skin reactions after CIRT have not been investigated. The present study aimed to identify these factors and evaluate the correlation between the severity of acute skin reactions and skin dose parameters.

**Methods:**

CIRT with total doses of 64.0–70.4 Gy (relative biological effectiveness [RBE]) was administered to 22 patients with MBSTs. The skin-tumor distance (STD), maximum skin total dose (Dmax), and area of the skin receiving a total dose of X Gy (RBE) were evaluated.

**Results:**

All patients developed acute skin reactions after CIRT, including Grades 1 and 2 dermatitis in 15 (71%) and 6 (29%) patients, respectively. There was a significant difference in the STD between the two groups (*P* = 0.007), and the cut-off value of STD for predicting Grade 2 acute skin reactions was 11 mm. There was a significant difference in Dmax between the groups (*P* < 0.001), and the cut-off value of Dmax for predicting Grade 2 acute skin reactions was 52 Gy (RBE). Significant differences between the two groups were observed in terms of the area irradiated with 40 Gy (RBE) (S40), and the cut-off value of S40 for predicting Grade 2 acute skin reactions was 25 cm^2^.

**Conclusions:**

In acute skin reactions after CIRT for MBSTs, STD, Dmax, and S40 were found to be significant predictive factors for acute skin reactions.

## Background

Malignant bone and soft tissue tumors (MBSTs) remain an intractable cancer. Recently, the clinical outcome of surgery for MBSTs was improved due to advancements in combined treatment with chemotherapeutic agents such as gemcitabine, vinorelbine, and docetaxel, and improvements in diagnostic radiology [1–4]. Consequently, the local recurrence rate for MBSTs is reported to be less than 20% when the tumors are resected with satisfactory surgical margins [5–7]. However, when the tumors are locally advanced and/or they are located near critical organs such as the spinal cord, resection with a satisfactory margin is often difficult, and the local recurrence rate remains as high as 50% [7–9]. Radiotherapy and/or chemotherapy are selected for the treatment of unresectable MBSTs. However, because MBSTs are relatively radioresistant, the local control rate of MBSTs treated with the conventional radiotherapy involving X-rays is relatively insufficient (14–56%) [8, 10, 11]. Furthermore, the treatment outcome of chemotherapy for MBSTs is poor [1–3]. Thus, the development of a novel treatment strategy for unresectable MBSTs is an urgent need.

Carbon ion radiotherapy (CIRT) has biological and physical advantages compared with the conventional radiotherapy with X-rays. Regarding the biological effect, the carbon ion beams have a relative biological effectiveness (RBE) that is 2–3-fold higher than that of X-rays [12, 13]. In addition, in terms of the physical aspect, the carbon ion beam is superior to X-rays, with regard to the sharpness of the dose distribution. This results from the ability of accelerated carbon ions to release a maximum amount of energy at the end of their track, resulting in a Bragg peak [14]. Because of the greater mass of carbon nuclei, the released energy exhibits a smaller amount of lateral scattering [14]. These features of the carbon ion beam permit dose escalation to be performed for tumors without increasing toxicity in the surrounding normal tissues. For such reasons, CIRT has been considered to possess high therapeutic potential for X-ray–resistant tumors. In fact, clinical trials studying CIRT for MBSTs were launched at the National Institute of Radiological Sciences (NIRS, Chiba, Japan) in 1996. A series of studies conducted at the NIRS reported favorable local control; specifically, the overall 3-year local control rate for MBSTs was 73% [15]. The 5-year local control rates were 86 and 62% in patients with unresectable pelvic chordoma and osteosarcoma of the trunk, respectively [16, 17]. These results suggest that CIRT is an effective local treatment for MBSTs and support its potential use as a first-line treatment in patients with unresectable tumors.

In CIRT for locally advanced MBSTs, the tumors are often located near the skin surface. Unlike X-rays, carbon ion beams have no build-up phenomenon; thus, as the worst-case scenario, the skin may receive the full prescribed dose at the entrance point of the irradiated beam, and severe adverse skin reactions may develop after CIRT. In a phase I/II dose escalation study on CIRT for patients with MBSTs that were not eligible for surgical resection, several severe skin reactions were reported at the highest dose level [15]. Therefore, the skin is considered a critical organ at risk in the treatment of CIRT for MBSTs.

Skin reactions after radiotherapy can be divided into acute and late phases. In radiotherapy using X-rays, acute skin reaction is a frequent adverse event that can be distressing to patients [18]. Moreover, every acute skin reaction can be followed by some degree of late or persistent skin reaction [18]. Accordingly, in CIRT for MBSTs, acute and late skin reactions are major concerns. Yanagi et al. analyzed late skin reactions in patients with MBST treated with CIRT using a dose-surface histogram (DSH) and identified ˃20 cm^2^ of skin receiving 60 Gy (RBE) as a significant predictor for severe late skin reactions (≥ Grade 3 using the Late Radiation Morbidity Scoring Scheme of the RTOG/EORTC) [19]. However, predictive dose-volume factors for acute skin reaction have not been explored. In the present study, to identify predictors of acute skin reaction induced by CIRT, we investigated the correlation between the severity of acute skin reactions and skin dose parameters, including dose-volume factors for patients with MBSTs treated with CIRT.

## Methods

### Patients

From November 2010 to December 2012, 22 consecutive patients with MBSTs were treated with CIRT at Gunma University Heavy Ion Medical Center (GHMC). The eligibility criteria for this study were as follows: (i) presence of a tumor histopathologically diagnosed as MBST; (ii) no lymph node metastasis or distant metastasis; (iii) tumor could be grossly measured using computed tomography (CT) or magnetic resonance imaging (MRI); (iv) age of 16–80 years; and (v) performance status of 0–3. Among the 22 patients, one patient was excluded from the analysis because two treatment positions (supine and prone) were used for treatment planning CT and accurate dose-surface analysis on composite dose was impossible. The characteristics of the patients investigated in the present study are summarized in Table 1. All patients were not eligible for curative total resection. Induction chemotherapy, concurrent chemotherapy or adjuvant chemotherapy within 90 days after CIRT was not performed in any patient. The study was approved by the institutional review board of Gunma University Graduate School of Medicine (approval number: 2016-042). Written informed consent was obtained from all patients.

**Table 1:** Patient characteristics

Sex	male: 16, female: 5
Age (years)	median 61.5 (19–79)
Tumor size (mm)	median 85 (52–126)
Tumor location	pelvis: 9, limbs: 4, trunk: 4, paravertebral: 3, peritoneal: 1
Pathological diagnosis	chordoma: 5, MFH: 3, chondrosarcoma: 3, lioposarcoma: 2, MPNST: 2, other: 5
Total dose (Gy(RBE])	64.0: 4, 67.2: 10, 70.4: 7

*MFH* malignant fibrous histiocytoma, *MPNST* malignant periferal nerve sheath tumor

### Carbon ion radiotherapy

The patients were positioned in customized cradles (Moldcare: Alcare, Tokyo, Japan) and immobilized using a low-temperature thermoplastic retaining device (Shellfitter: Kuraray, Tokyo, Japan). Patients were placed in the supine and prone position. Although the rotating gantry system for CIRT was not installed at GHMC, to reduce the dosage of organs at risk including the skin, irradiation was delivered from multiple directions using a rotational couch. In such cases, in addition to the spine and prone position, the cradle could be tipped −20 to 20 degrees to ensure that the beam is pointed in the appropriate direction, which will spare the organs at risk via the lateral edge of the beam. Furthermore, the treatment plan was modified according to tumor shrinkage during the CIRT treatment duration. A set of CT images with 2-mm–thick slices was taken for treatment planning with the same posture of the patients retained in the immobilization devices. For treatment planning, a respiratory-gated CT scan was performed near the end of the expiration phase.

Patients were irradiated using passive broad beam methods. Treatment planning was performed using the XiO-N system (Elekta AB, Stockholm, Sweden). The gross tumor volume (GTV) was delineated on the CT images with reference to MRI and/or positron emission tomography with 2-deoxy-2-[fluorine-18]-fluoro-d-glucose. The clinical target volume (CTV) included the GTV and tissues at risk of microscopic involvement. The planning target volume (PTV) consisted of the CTV, the internal margin, and a set-up margin corresponding to the sum of the error lengths at positioning. The target reference point was located at the isocenter, and the PTV was covered by ≥ 95% of the prescribed dose. Generally, the total dose was set at 70.4 Gy (RBE). For chordoma, the total dose was set at 67.2 Gy (RBE), based on a previous report [16]. In patients in whom the tumors were located close to the spinal cord, the total dose was set at 64.0 Gy (RBE) [17]. CIRT was administered once daily 4 days a week over 4 weeks. One port was used in 1 session per day, and treatment was performed on 4 consecutive days per week from Tuesday to Friday. The PTV margin was modified for patients in whom the tumors were located close to critical organs such as the bowel and skin. The limiting doses for the bowel and skin were defined as D2cc < 44 Gy (RBE) and ≤ 20 cm^2^ of skin receiving 60 Gy (RBE), respectively, to avoid severe late reactions [19]. Carbon ion beams with energy levels of 290, 350, and 400 MeV were used.

### Evaluation of the skin reaction

In the present study, the correlation between acute skin reactions after CIRT and skin dose parameters was investigated. Photographs of the irradiated skin were obtained at least once a week during CIRT as well as 2 and 3 months after the completion of CIRT. In every photograph, radiation therapy-induced dermatitis was graded using the Common Terminology Criteria for Adverse Events version 4.0 by two radiation oncologists (Y.T. and H.K.). In each patient, the maximum grade scored for acute (≤ 90 days) and late skin reactions (> 90 days) during the follow-up period was evaluated.

### Parameters for acute skin reaction

In acute skin reactions, the effect of ionizing irradiation on the epidermis is considered pivotal [20]. The average thickness of the epidermis is 0.2 mm [21]. In the present study, based on these findings, we defined the region within 0.2 mm under the skin surface as “the skin” value. The average of the doses delivered to the skin was used in the analysis. In cases in which the treatment was performed in multiple positions, the skin dose was calculated after fusing the treatment plans in these positions.

In the present study, three parameters for the skin were employed: (i) the skin-tumor distance (STD), which was the minimum distance between the GTV and the skin surface; (ii) maximum total irradiation dose that the skin received (Dmax); and (iii) the area of the skin receiving a total dose of X Gy (RBE), described as SX [19]. The analyses were performed using MIM maestro software version 5.6. (MIM Software Inc. Cleveland, USA) [22].

### Statistical analysis

Significant differences were analyzed using the unpaired two-tailed Student’s *t*-test with STSS software version 5.0 (SAS Institute, Cary, NC, USA). *P* < 0.05 was considered significant. Receiver operating characteristic (ROC) curves were generated and used to determine the optimal cut-off value.

## Results

### Skin reaction

All patients developed acute skin reactions after CIRT. Among them, 15 (71%) and 6 (29%) patients developed Grades 1 and 2 dermatitis, respectively. No patients experienced ≥ Grade 3 acute radiation dermatitis, and 15 developed Grade 1 late skin reactions. No patients experienced ≥ Grade 2 severe late radiation dermatitis during a median follow-up period of 42.5 months.

### Skin-tumor distance

**Fig. 1:**
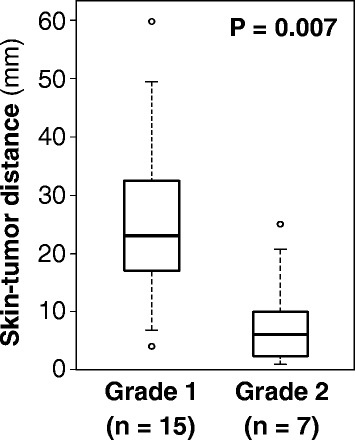
Skin-tumor distance (STD). The median STDs in the Grades 1 and 2 groups were 23 (range, 4.0–60) and 5 (range, 1–25) mm, respectively (P = 0.007). The cut-off value for predicting Grade 2 acute skin reactions was 11 mm (sensitivity = 86%, specificity = 87%)

The STD in patients who exhibited Grades 1 and 2 acute skin reactions is summarized as a boxplot (Fig. 1). The median STD in all patients was 18 (range, 1.0–60) mm, whereas those in the Grades 1 and 2 groups were 23 (range, 4.0–60) and 5 (range, 1–25) mm, respectively. There was a significant difference in the STDs between patients in the Grade 1 and 2 groups (*P* = 0.007). Using ROC curve analysis, the cut-off value was determined to be 11 mm for predicting Grade 2 acute skin reactions; at this value, the sensitivity and specificity were calculated as 86 and 87%, respectively.

### Skin maximum dose

**Fig. 2:**
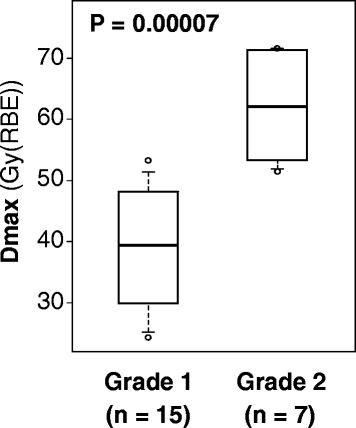
Maximum skin total dose (Dmax). The median Dmax values in the Grades 1 and 2 groups were 39 (range, 24–53) and 62 (range, 52–72) Gy (RBE), respectively (*P* < 0.001). The cut-off value was 52 Gy (RBE) (sensitivity = 100%, specificity = 87%)

The Dmax data for patients with Grades 1 and 2 acute skin reactions are summarized as a boxplot (Fig. 2). The median Dmax in all patients was 48 (range, 24–72) Gy (RBE), whereas those in the Grades 1 and 2 groups were 39 (range, 24–53) and 62 (range, 52–72) Gy (RBE), respectively. There was a significant difference in the Dmax between the Grades 1 and 2 groups (*P* < 0.001). The ROC curve analysis revealed a cut-off value of 52 Gy (RBE) for the prediction of Grade 2 acute skin reactions, at which the sensitivity and specificity were calculated as 100 and 93%, respectively.

### Dose-surface histogram analysis

**Fig. 3:**
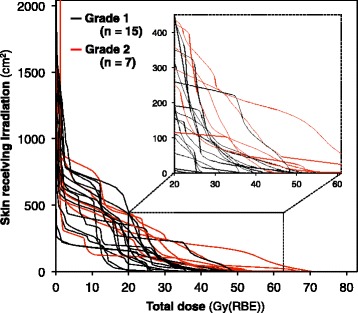
Dose-surface histogram in all patients. The Grade 2 group (red line) tended to receive a higher dose in a wider skin area than the Grade 1 group (black line)

**Fig. 4:**
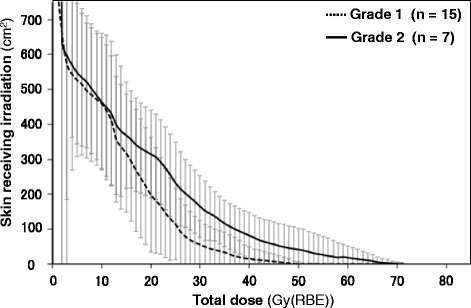
The average dose-surface histogram in the Grades 1 and 2 groups. These data illustrated the differences between these two groups and indicated that the Grade 2 group received a high radiation dose

**Fig. 5:**
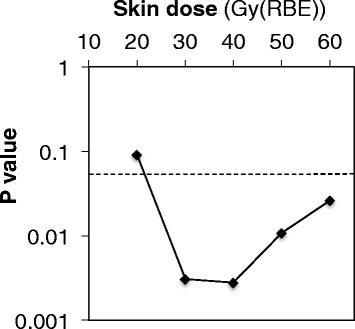
*P* value of the area of the skin receiving a total dose of X Gy (Sx). Significant differences between the two groups were observed in terms of S30, S40, S50, and S60, with the lowest P value observed for S40 (P = 0.002)

**Fig. 6:**
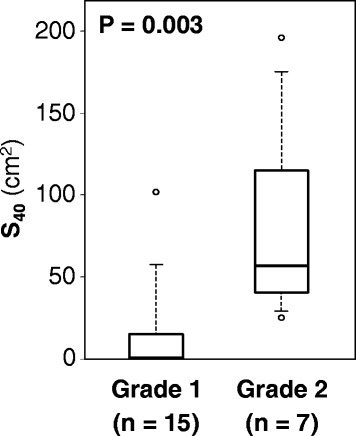
Area of the skin receiving a total dose of 40 Gy (S). The median S_40_ values in the Grades 1 and 2 groups were 0 (range, 0–102) and 57 (range, 25–196) cm^2^, respectively (*P* < 0.05). The cut-off value was 25 cm^2^ (sensitivity = 100%, specificity = 80%)

A DSH demonstrating the relationship between the total radiation dose to the skin and the irradiated skin area in all the patients is shown in Fig. 3. The DSH indicated that the patients in the Grade 2 group tended to receive a higher dose in a wider skin region than those in the Grade 1 group. The average DSH for the Grades 1 and 2 groups regarding acute skin reactions are presented in Fig. 4. The average DSH data illustrated a clear difference between these two groups at moderate and high doses and indicated that the Grade 2 group received a high radiation dose. The statistical significance concerning SX between the groups was assessed where X ranged from 0 to 60 Gy (RBE) at increments of 10 Gy (RBE) (Fig. 5). Consequently, significant differences between the two groups were observed in terms of S30, S40, S50, and S60, with the lowest *P* value observed for S40 (*P* = 0.002). S40 in the Grades 1 and 2 groups is summarized as a boxplot (Fig. 6). The median S40 in all patients was 14.2 (range, 0.0–196.0) cm^2^, and those in the Grades 1 and 2 groups were 0 (range, 0–102) and 57 (range, 25–196) cm^2^, respectively. The ROC curve analysis determined a cut-off value of 25 cm^2^ for the prediction of Grade 2 acute skin reactions, at which the sensitivity and specificity were calculated as 100 and 80%, respectively.

## Discussion

In the present study, severe acute skin reactions greater than Grade 3 were not observed. We have complied the dose constrain for late skin reaction [15] by using multiple beam directions and a lateral edge technique of the carbon ion beams to spare the skin. As the severity of acute skin reactions generally correlates with that of late skin reactions [15], it is believed that efforts to avoid severe late skin reactions helped to reduce the incidence of severe acute reactions. Although only Grades 1 and 2 acute skin reactions were analyzed in the present study, the STD, skin maximum dose, and S40 were prognostic factors associated with the acute skin reaction. To our knowledge, the present study is the first report on the relationship between acute skin reactions and skin dose parameters in CIRT for MBSTs.

Acute skin reactions are frequent adverse events of radiotherapy, and they can be distressing to patients [18]. In CIRT in particular, acute and late skin reactions may be more serious when the full prescription dose is delivered to a large area of the skin. In a phase I/II dose escalation study of CIRT for MBSTs, eight patients developed Grade 3 acute skin reactions and four developed Grade 4 late skin reactions at a dose level of 73.6 Gy (RBE) delivered in 16 fractions over 4 weeks [15]. This study concluded that the maximum tolerated dose was 73.6 Gy (RBE). Consequently, the recommended dose was set at 70.2, 67.2, or 64.0 Gy (RBE) according to the types of MBSTs, and treatment planning was optimized using a DSH model. Since then, the incidence of Grade 4 late skin reaction has decreased [16]. In addition to the previous treatment strategy at NIRS, our findings provided information on the dose-response relationship between acute skin reactions and skin dose parameters.

Lienger et al. reported the relationship between the X-ray radiation dose and acute skin reactions [23]. A total dose of 35–45 Gy delivered to a 30–150-cm^2^ field led to erythema, and 40–65 Gy delivered to a 230–420-cm^2^ field resulted in worse acute skin reactions [23]. Regarding late skin reactions after X-ray irradiation, Emami et al. reported that treatment with a total dose of 60 Gy delivered to a 30-cm^2^ field, or 70 Gy delivered to a 10-cm^2^ field increased a probability of 5% skin necrosis within 5 years [24]. Li-Min et al. reported the relationship between acute skin reactions and X-ray radiotherapy for breast cancer [25]. The authors proposed that patients with larger breast volumes (≥ 350 ml) had a significant higher risk of developing moist desquamation. These studies demonstrated that skin reactions were dependent on the dose and volume.

The skin has been recognized as a critical organ at risk at the start of modern radiotherapies such as stereotactic body radiation therapy, intensity-modulated radiotherapy (IMRT), and proton therapy. Hoppe et al. reported that in stereotactic radiation therapy for stage I lung cancer, predictive factors associated with Grade 2 or higher acute skin reactions included the usage of only three beams, a distance of less than 5 cm between the tumor and the posterior chest wall skin, and a maximum skin dose of 50% or more of the prescribed dose [26]. As noted in the present study, it was suggested that STD and Dmax were correlated with the severity of skin reactions. Galland et al. prospectively compared skin toxicities after proton therapy for breast cancer with those of photon-based 3-dimensional conformal accelerated partial breast irradiation [27]. The incidences of telangiectasia, pigmentation change, and other late skin toxicities were significantly higher in the proton therapy group, which necessitated the use of multiple-field or scanning techniques to minimize skin toxicities. Similarly, in CIRT, careful treatment planning and evaluation for skin reaction before treatment are important. In the present study, to prevent severe skin reactions among patients for whom the tumor was located near the skin, the CIRT treatment plan was devised to reduce the skin dose by using techniques such as sparing the skin via the lateral edge of the beam, multiple directions for the carbon ion beam, and modifying the treatment plan according to tumor shrinkage.

In conventional X-ray radiotherapy, skin irradiation has been described in terms of the field size and prescription dose [23, 24]. However, a consensus has not yet been achieved regarding the surface- or volume-based evaluation method for skin irradiation in X-ray radiotherapy, proton therapy, and CIRT. Although one study on IMRT evaluated the skin dose in patients with head and neck cancer, detailed information about the definition of the skin was not provided [18]. Yanagi et al. calculated the skin dose using voxels [19]. As the epidermis plays a leading role in acute skin reactions, we defined the region within 0.2 mm of the skin surface, which is the average thickness of the epidermis, as the skin. In this manner, dose-response relationships concerning acute skin reactions were observed. A sequential study concerning the validity of the current dose assessment method is required.

Despite limitations such as the small sample size and the absence of severe acute skin reactions, a significant correlation was observed between acute skin reactions and skin dose parameters in CIRT for MBSTs. The cut-off values of the dose parameters in the present prospective study will be useful not for determining dose constraints for the skin, but for optimizing treatment planning in balance with tumor coverage. Further observation with a large patient cohort will be necessary to confirm our findings.

## Conclusions

In summary, STD, Dmax, and S40 were found to be significant predictive factors for acute skin reactions after CIRT for MBSTs. The present study will be useful for optimizing treatment planning in CIRT for MBSTs.

## Abbreviations

CIRT: Carbon ion radiotherapy
CTV: Clinical target volume
Dmax: maximum skin total dose
DSH: Dose-surface histogram
GHMC: Gunma University Heavy Ion Medical Center
GTV: Gross tumor volume
IMRT: Intensity-modulated radiotherapy
MBSTs: Malignant bone and soft tissue tumors
NIRS: National Institute of Radiological Sciences
PTV: Planning target volume
RBE: Relative biological effectiveness
ROC: Receiver operating characteristic
STD: Skin-tumor distance
